# Prognostic risk classification for biochemical relapse-free survival in patients with oligorecurrent prostate cancer after [^68^Ga]PSMA-PET-guided metastasis-directed therapy

**DOI:** 10.1007/s00259-020-04760-8

**Published:** 2020-03-16

**Authors:** Marco M. E. Vogel, Stephanie G. C. Kroeze, Christoph Henkenberens, Nina-Sophie Schmidt-Hegemann, Simon Kirste, Jessica Becker, Irene A. Burger, Thorsten Derlin, Peter Bartenstein, Michael Mix, Christian la Fougère, Matthias Eiber, Hans Christiansen, Claus Belka, Anca L. Grosu, Arndt-Christian Müller, Matthias Guckenberger, Stephanie E. Combs

**Affiliations:** 1 Department of Radiation Oncology, Klinikum rechts der Isar, Technical University of Munich (TUM), Ismaninger Strasse 22, 81675 Munich, Germany; 2 Department of Radiation Sciences (DRS), Institute for Radiation Medicine (IRM), Helmholtz Zentrum München, Neuherberg, Germany; 3 Department of Radiation Oncology, University Hospital Zurich, University of Zurich, Zurich, Switzerland; 4 Department of Radiotherapy and Special Oncology, Medical School Hannover, Hannover, Germany; 5 Department of Radiation Oncology, University Hospital LMU Munich, Munich, Germany; 6 Department of Radiation Oncology, Medical Center University of Freiburg, Faculty of Medicine, University of Freiburg, Freiburg, Germany; 7 German Cancer Consortium (DKTK), Partner Site Freiburg, Freiburg, Germany; 8 Department of Radiation Oncology, University Hospital Tübingen, Eberhard Karls University Tübingen, Tübingen, Germany; 9 Department of Nuclear Medicine, University Hospital Zurich, University of Zurich, Zurich, Switzerland; 10 Department of Nuclear Medicine, Hannover Medical School, Hannover, Germany; 11 Department of Nuclear Medicine, University Hospital LMU Munich, Munich, Germany; 12 Department of Nuclear Medicine, Medical Center University of Freiburg, Faculty of Medicine, University of Freiburg, Freiburg, Germany; 13 Department of Nuclear Medicine and Clinical Molecular Imaging, University Hospital Tübingen, Eberhard Karls University Tübingen, Tübingen, Germany; 14 German Cancer Consortium (DKTK), Partner Site Tübingen, Tübingen, Germany; 15 Cluster of Excellence iFIT (EXC 2180) “Image Guided and Functionally Instructed Tumor Therapies”, University of Tübingen, Tübingen, Germany; 16 Department of Nuclear Medicine, Klinikum rechts der Isar, Technical University of Munich (TUM), Munich, Germany; 17 German Cancer Consortium (DKTK), Partner Site Munich, Munich, Germany

**Keywords:** Prostate-specific membrane antigen-positron emission tomography, Prostatic carcinoma, Oligometastatic, Oligorecurrent, MDT

## Abstract

**Purpose:**

Since the success of prostate-specific membrane antigen-positron emission tomography (PSMA-PET) imaging for patients with oligorecurrent prostate cancer (ORPC), it is increasingly used for radiotherapy as metastasis-directed therapy (MDT). Therefore, we developed a prognostic risk classification for biochemical relapse-free survival (bRFS) for patients after PSMA-PET-guided MDT after radical prostatectomy.

**Methods:**

We analyzed 292 patients with local recurrence (LR) and/or pelvic lymph node (LN) lesions and/or up to five distant LN, bone (BM), or visceral metastases (VM) detected with [^68^Ga]PSMA-PET imaging. Median follow-up was 16 months (range 0–57). The primary endpoint was bRFS after MDT. Cox regression analysis for risk factors was incorporated into a recursive partitioning analysis (RPA) with classification and regression tree method.

**Results:**

PSA at recurrence ≥ 0.8 ng/mL, BM, and VM was significantly associated with biochemical relapse. RPA showed five groups with tenfold cross-validation of 0.294 (SE 0.032). After building risk classes I to IV (*p* < 0.0001), mean bRFS was 36.3 months (95% CI 32.4–40.1) in class I (PSA < 0.8 ng/mL, no BM) and 25.8 months (95% CI 22.5–29.1) in class II (PSA ≥ 0.8 ng/mL, no BM, no VM). LR and/or pelvic LNs caused relapse in classes I and II. Mean bRFS was 16.0 months (95% CI 12.4–19.6) in class III (PSA irrelevant, present BM) and 5.7 months (95% CI 2.7–8.7) in class IV (PSA ≥ 0.8 ng/mL, no BM, present VM).

**Conclusion:**

We developed and internally validated a risk classification for bRFS after PSMA-PET-guided MDT. Patients with PSA < 0.8 ng/mL and local relapse only (LR and/or pelvic LNs) had the most promising bRFS. PSA ≥ 0.8 ng/mL and local relapse only (LR and/or pelvic LNs) indicated intermediate risk for failure. Patients with BM were at higher risk regardless of the PSA. However, those patients still show satisfactory bRFS. In patients with VM, bRFS is heavily decreased. MDT in such cases should be discussed individually.

## Introduction

In 1995, Weichselbaum et al. hypothesized that an oligometastatic state in cancer progression exists [1]. On that basis, the oligorecurrent paradigm was also introduced into prostate cancer (PC) management. However, several descriptions of oligometastatic PC exist, and definitions vary between less than three to five distant lesions [2]. Data show a better outcome for patients with oligometastatic PC than for patients with advanced metastatic disease [3]. Recently, Ost et al. demonstrated that patients with oligorecurrent PC treated with metastasis-directed therapy (MDT) had improved freedom from androgen deprivation therapy (ADT) (13 vs. 21 months) [4]. Furthermore, Decaestecker et al. reported a prolonged time to ADT and no grade III toxicity after repeated stereotactic body radiotherapy (SBRT) [5]. Interestingly, even by using less sensitive choline positron emission tomography (PET) imaging for staging in the trials mentioned above [4, 5], a good response to MDT was observed.

Nevertheless, standard-of-care treatment for oligorecurrent PC remains empiric salvage radiotherapy (RT) for suspect local recurrence in the prostatic bed or ADT for patients with distant metastases. Due to limited treatment and diagnostic options, local therapy of the metastatic sites has been rarely used in the past. With the increasing application of prostate-specific membrane antigen (PSMA)-PET imaging, the treatment of patients with few metastases has changed. PSMA-PET imaging has become an effective tool for staging and defining targets for precise treatment of patients with biochemical relapse after radical prostatectomy (RP) [6, 7]. Whereas in the past, radiation oncologists had to administer empiric treatment to the prostate bed mostly without evidence in imaging; today, PSMA-PET helps to visualize recurrent tumor sites and distant lesions and provides a highly sensitive means for estimating the accurate tumor load.

PSMA-PET imaging enriches the possibilities of MDT with conventional fractionated RT, stereotactic body RT (SBRT), or surgery. These can prolong time to initiation of ADT [2] and bear the possibility of cure.

Up to now, the optimal selection of patients for MDT is unknown. The majority of patients will develop progression, despite PSMA-PET staging and MDT. Therefore, clinical risk classifications have become an accepted instrument for weighing the risk-benefit ratio. To our knowledge, no such tools have been published for patients with oligorecurrent PC treated with MDT using highly sensitive PSMA-targeted imaging. In the present analysis, we aimed to develop a prognostic risk classification predicting biochemical relapse-free survival (bRFS) after PSMA-PET-guided MDT for oligorecurrent prostate cancer after prior RP.

## Material and methods

### Patients

**Fig. 1:**
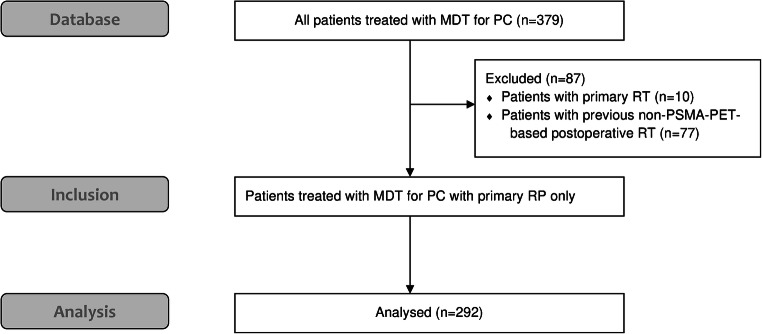
Flow diagram of included patients (MDT, metastasis-directed therapy; PC, prostate cancer; RP, radical prostatectomy; RT, radiotherapy)

We established a multi-institutional, retrospective database, and collected data of 379 patients from six German and Swiss centers. The institutional review boards of all participating centers approved the study (BASEC-Nr. 2017-01499). The subjects were free from distant metastases (M0) at initial diagnosis. We defined the relapse as biochemical failure and recurrence diagnosed by PSMA-PET imaging. The patients exhibited oligorecurrent disease with local recurrence and/or pelvic lymph node lesions (N1) and/or distant metastases (M1a/1b/1c) in PSMA-PET imaging. We included patients with up to five distant lymph node, bone, or visceral lesions and any PSA level at PSMA-PET-based diagnosis. There was no restriction on the total number of pelvic lymph node lesions. The database included patients treated with MDT for PC metastases independently of the primary therapy. In the present analysis, we included 292 patients with initial RP and a subsequent diagnosis of oligorecurrent PC with positive findings in PSMA-PET imaging. Patients with other primary therapy for PC (e.g., primary RT) or with previous postoperative non-PSMA-PET-based RT were exclude from this analysis (*n* = 87). The flow diagram is shown in Fig. 1.

### PSMA-PET imaging and RT

Each patient received PET imaging with a [^68^Ga]-labeled PSMA targeting ligand [8]. Imaging was performed according to the joint EANM and SNMMI guidelines [9]. PSMA-PET imaging was acquired in conjunction with either computed tomography (CT) or magnetic resonance imaging (MRI). The CT was performed as a diagnostic CT or as a low-dose CT. Intravenous and if necessary oral iodinated contrast agents were used if the patients had no contraindications. CT scans were acquired in the portal venous phase. MRI scans where performed with contrast agents, when possible. PET scans were conducted approximately 60 min after bolus injection of the ligand (mean activity 162.5 MBq; range 87.0–291.0 MBq). When possible, furosemide 20 mg was given to reduce tracer collection in the urinary tract system. One specialist in nuclear medicine and one radiologist or a dual boarded nuclear medicine physician and radiologist interpreted the acquired imaging. Focal tracer uptake higher than the surrounding background and not associated with physiologic uptake was considered as malignant.

MDT as conventional fractionated RT or SBRT was performed at the discretion of the study centers. Furthermore, ADT was administered according to the guidelines of the participating departments.

### Endpoints

The primary endpoint was bRFS, which was measured from MDT to biochemical relapse. We defined biochemical relapse as PSA levels ≥ 0.2 ng/mL above PSA nadir after RT. When PSA levels did not respond to RT, pre-RT PSA level with a rise of ≥ 0.2 ng/mL was defined as a relapse. Follow-up was performed according to institutional protocol.

### Statistical analysis

We applied univariate and multiple Cox regression to assess significance for risk factors for biochemical relapse. We evaluated initial tumor stage [10] (T2c ≤ vs. ≥ T3a), initial nodal status [11] (N0 vs. N1), initial Gleason score [10, 11] (7a ≤ vs. ≥ 7b), and initial resection status [11] (R0 vs. R1). Further, we evaluated the risk factors PSA persistence > 0.1 ng/mL after RP [12], PSA level at PMSA-PET imaging/recurrence [10, 11], age at relapse as well as the PSMA-PET-related factors local recurrence (no vs. yes), pelvic lymph node lesions (no vs. yes), distant metastases (no vs. yes), and the number of lesions at PSMA-PET imaging. We used receiver operating characteristic (ROC) analysis to determine cutoff values for non-dichotomous variables. To account for the possible confounder of additive ADT administration and to show robustness of the model, multiple Cox regression was calculated unadjusted and adjusted for additive ADT. Risk factors which showed significance in both models were included into the risk classification. We used recursive partitioning analysis (RPA) with the classification and regression tree (CRT) method to analyze the significant factors. bRFS at 12 months was the primary endpoint in the decision tree calculation. Decision tree analysis is a nonparametric automatic statistical learning algorithm that combines variables. The results are shown in a decision tree with splits based on the variables. The Gini index was applied to minimize node impurity after splitting. End nodes of the decision tree were grouped from A to E. For internal validation, we used tenfold cross-validation. In the tenfold cross-validation scheme, patients were randomly assigned into ten equal subgroups. Nine subsets were utilized as a training cohort. The tenth group was used as a validation set to test the performance. Subsequently, risk classes I to IV (low to very high risk) were built by combining end node groups with a similar outcome in Kaplan-Meier estimator for the overall group. We used the Kaplan-Meier method to calculate bRFS of the risk classes. All statistical analyses were performed with SPSS version 21 (IBM, Armonk, USA). A *p* value of less than 0.05 was defined as statistically significant.

## Results

All patients were treated for oligorecurrent PC with conventional fractionated RT and/or SBRT between April 2013 and January 2018. The median age of all patients was 70 years (range 46–95 years). Median follow-up was 16 months (range 0–57 months). Table 1 shows the patient characteristics, and Table 2 shows the numbers of biochemical relapse and local control.

**Table 1:** Patient characteristics (*n* = 292)

	All patients (*n* = 292) *n* (%)
Initial tumor classification	
pT1c	1 (0.3%)
pT2a	9 (3.1%)
pT2b	10 (3.4%)
pT2c	96 (32.9%)
pT3a	71 (24.3%)
pT3b	96 (32.9%)
pT4	9 (3.1%)
Initial nodal status	
Negative	185 (63.4%)
Positive	95 (32.5%)
Missing	12 (4.1%)
Gleason score	
6	16 (5.5%)
7a	67 (22.9%)
7b	86 (29.5%)
≥ 8	123 (42.1%)
Surgical margin	
Negative	161 (55.1%)
Positive	131 (44.9%)
Parameters at PSMA-PET-based diagnosis of oligorecurrent disease	
Median age at PSMA-PET imaging (years)	70 (range 46–95)
Median PSA level at PSMA-PET imaging (ng/mL)	0.95 (range 0.04–40.13)
Local recurrence at PSMA-PET imaging	
No	163 (55.8%)
Yes	129 (44.2%)
Pelvic lymph node metastases at PSMA-PET imaging	
Negative	135 (46.2%)
Positive	157 (53.8%)
Distant metastases at PSMA-PET imaging	
Lymph nodes (M1a)	
No	267 (91.4%)
Yes	25 (8.6%)
Bone (M1b)	
No	247 (84.6%)
Yes	45 (15.4%)
Visceral (M1c)	
No	289 (99.0%)
Yes	3 (1.0%)
Median total number of PET-positive lesions	1 (range 1–19)
Pelvic lymph node lesions	1 (range 1–19)
Distant metastases	1 (range 1–5)
RT Technique	
Conventional fractionated RT	265 (90.8%)
SBRT	17 (5.8%)
Conventional fractionated RT + SBRT	10 (3.4%)
Elective RT to prostate bed or locoregional lymph nodes	
No	72 (24.7%)
Yes	220 (75.3%)
Median EQD2/1.5 Gy (Gy)	
Prostatic fossa	66.0 (range 47.5–70.0)
Pelvic lymphatic pathways	47.5 (range 42.0–56.0)
PET-positive local recurrence	70.0 (range 57.7–83.0)
PET-positive lymph nodes	60.0 (range 46.0–85.0)
Bone lesions	56.0 (range 40.0–108.6)
Visceral lesions	108.6 (range 103.0–162.0)
Additive ADT	
No	178 (61.0%)
Yes	114 (39.0%)
Median follow-up (months)	16 (range: 0–57)

*PSMA-PET*, prostate-specific membrane antigen-positron emission tomography; *PSA*, prostate-specific antigen; *RT*, radiotherapy; *SBRT*, stereotactic body radiotherapy; *EQD2/1.5Gy*, equivalent dose in 2 Gy for alpha/beta of 1.5 Gy; *ADT*, androgen deprivation therapy

**Table 2:** Number of biochemical relapse and in-field local control

Biochemical relapse	
Biochemical relapse at 12 months	
Yes	19.5% (57/292)
No	47.9% (140/292)
No information	2.1% (6/292)
Censored	30.5% (89/292)
Overall biochemical relapse	
Yes	29.8% (87/292)
No	68.1% (199/292)
No information	2.1% (6/292)
In-field local control	
Local recurrence	96.4% (53/55), missing information on 40 lesions
Pelvic lymph nodes	96.0% (145/151), missing information on 48 lesions
Bone lesions	100% (32/32), missing information on nine lesions
Visceral lesions	100% (2/2), missing information on one lesion

Patients with missing information on lesions received no imaging due to no biochemical relapse

Table 3 shows the results of the univariate Cox regression of prognostic factors affecting bRFS. ROC analysis determined 0.8 ng/mL as the significant cutoff value for PSA before RT. A PSA persistence after RP ≥ 0.1 ng/mL, PSA levels prior to RT ≥0.8 ng/mL, the presence of bone metastases, and the presence of visceral lesions were significantly associated with relapse in the univariate analysis. Multiple Cox regression unadjusted and adjusted for additive ADT is shown in Table 4. PSA levels ≥ 0.8 ng/mL, the presence of bone metastases, and the presence of visceral metastases remained significant factors in both models.

**Table 3:** Univariate Cox regression of prognostic factors for biochemical relapse

Factors	Univariate Cox regression		
	HR	95% CI	*p*
Initial tumor stage (T2c ≤ vs. ≥ T3a)	0.67	0.43–1.05	0.08
Initial nodal status (N0 vs. N1)	0.67	0.43–1.05	0.08
Initial Gleason score (7a ≤ vs. ≥ 7b)	0.66	0.39–1.08	0.10
Initial resection status (R0 vs. R1)	1.02	0.67–1.58	0.91
PSA persistence > 0.1 ng/mL after RP (yes vs. no)	1.59	1.04–2.43	0.03*
PSA level at PMSA-PET imaging (> 0.8 ng/mL vs. ≤ 0.8 ng/mL)	0.51	0.32–0.79	0.003*
Age at relapse (continuous)	1.00	0.98–1.03	0.90
Local recurrence at PSMA-PET imaging (no vs. yes)	1.46	0.95–2.26	0.09
Pelvic lymph node lesions at PSMA-PET imaging (no vs. yes)	1.05	0.69–1.60	0.82
Distant metastases at PSMA-PET imaging			
Lymph nodes (no vs. yes)	0.80	0.40–1.60	0.53
Bone (no vs. yes)	0.38	0.24–0.62	0.0001*
Visceral (no vs. yes)	0.19	0.05–0.78	0.02*
Total number of lesions (1 versus > 1)	0.96	0.58–1.59	0.87
Use of additive ADT	2.56	1.51–4.36	0.0005*

*PSA*, prostate-specific antigen; *RP*, radical prostatectomy; *PSMA-PET*, prostate-specific membrane antigen-positron emission tomography; *ADT*, androgen deprivation therapy; *HR*, hazard ratio; *95% CI*, 95% confidence interval

*Significant result

**Table 4:** Multiple Cox regression of prognostic factors for biochemical relapse unadjusted and adjusted for additive androgen deprivation therapy

Factors	Multiple Cox regression unadjusted for ADT			Multiple Cox regression adjusted for ADT		
	HR	95% CI	*p*	HR	95% CI	*p*
PSA persistence > 0.1 ng/mL after RP (yes vs. no)	1.51	0.98–2.35	0.07	1.78	1.14–2.77	0.011*
PSA level at PMSA-PET imaging (> 0.8 ng/mL vs. ≤ 0.8 ng/mL)	0.62	0.39–0.98	0.04*	0.50	0.31–0.79	0.003*
Distant bone metastases at PSMA-PET imaging (no vs. yes)	0.39	0.24–0.64	0.0002*	2.17	1.33–3.56	0.002*
Distant visceral metastases at PSMA-PET imaging (no vs. yes)	0.09	0.02–0.38	0.001*	7.65	1.75–33.44	0.007*
Use of additive ADT	–	–	–	3.24	1.87–5.60	0.00002*

*PSA*, prostate-specific antigen; *RP*, radical prostatectomy; *PSMA-PET*, prostate-specific membrane antigen-positron emission tomography; *ADT*, androgen deprivation therapy; *HR*, hazard ratio; *95% CI*, 95% confidence interval

*Significant result

**Fig. 2:**
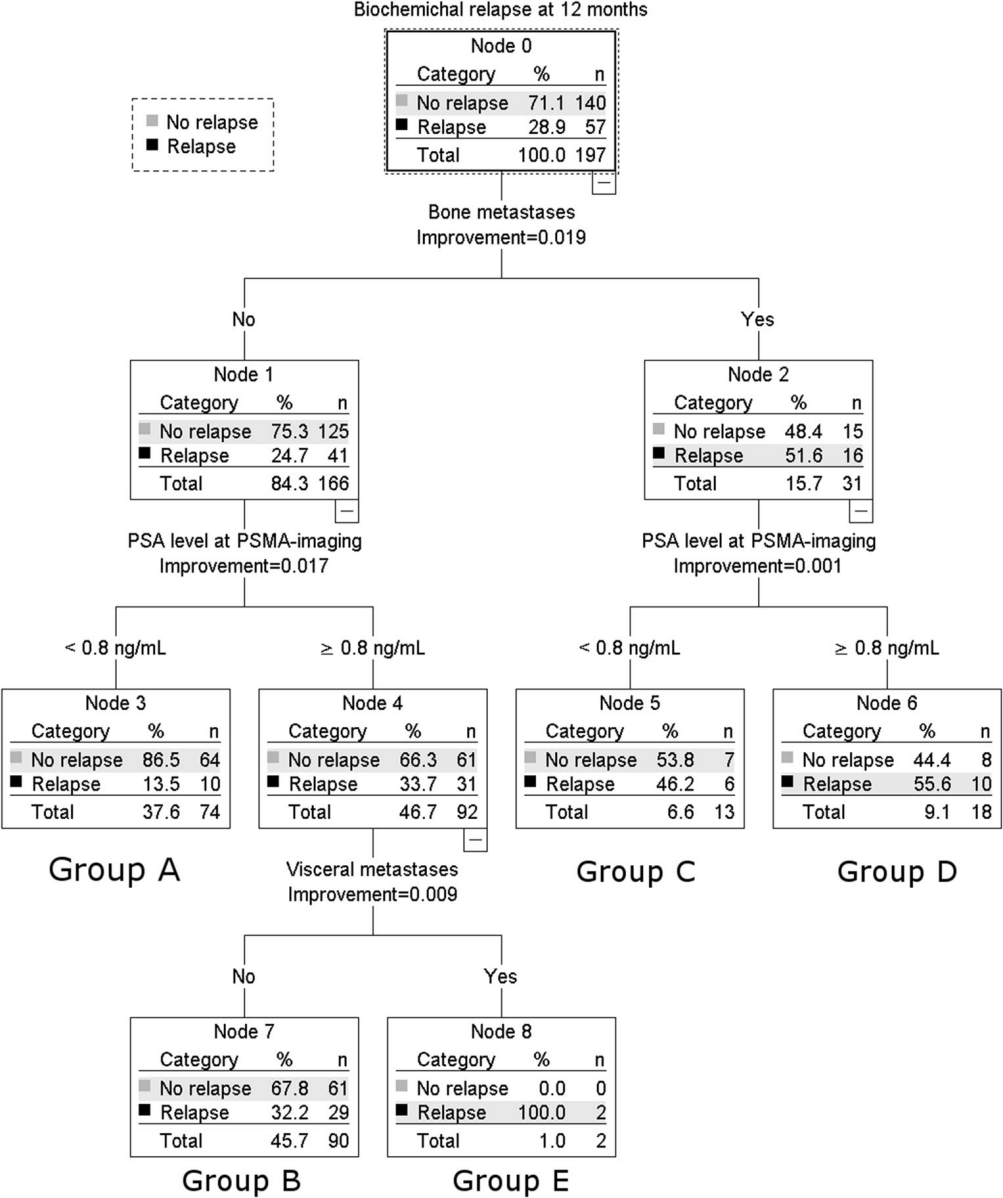
Recursive partitioning analysis (RPA) with the classification and regression tree (CRT) method (*n* = 197, data of 95 patients was censored at 12 months). End nodes are marked with groups A to E. (PSMA-PET, prostate-specific membrane antigen-positron emission tomography; PSA, prostate-specific antigen; ng/mL, nanogram/milliliter)

We included the three significant factors of both models into the RPA using the CRT method. Figure 2 shows the decision tree with the end nodes A to E. Tenfold cross-validation demonstrated a risk for miscalculation of 0.294 (standard error 0.032), which results in 70.6% accuracy.

**Fig. 3:**
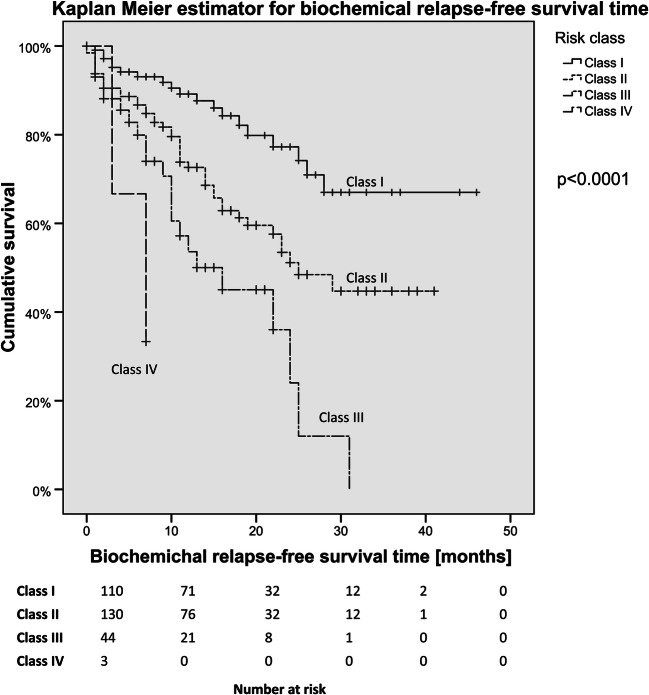
Kaplan-Meier estimator for biochemical relapse-free survival time in months stratified for risk classes I to IV for the overall group

The Kaplan-Meier estimator showed a mean bRFS of 36.3 months (95% CI 32.4–40.1 months) for group A (PSA < 0.8 ng/mL, no bone lesions), of 25.8 months (95% CI 22.5–29.1 months) for group B (PSA ≥ 0.8 ng/mL, no bone or visceral lesions), of 15.6 months (95% CI 11.3–20.0 months) for group C (PSA < 0.8 ng/mL, present bone lesions), of 16.6 months (95% CI 11.2–22.0 months) for group D (PSA ≥ 0.8 ng/mL, present bone lesions), and of 5.7 months (95% CI 2.7–8.7 months) for group E (PSA ≥ 0.8 ng/mL, no bone lesions, present visceral lesions). Subsequently, we built risk classes with a similar outcome: risk class I with low risk (group A), class II with intermediate risk (group B), class III with high risk (groups C and D), and class IV with very high risk (group E). We used the Kaplan-Meier estimator to compare survival among risk classes for the overall group (see Fig. 3). The log-rank test showed significant differences among groups with *p* < 0.0001.

The risk classification showed that patients with PSA levels < 0.8 ng/mL and no bone metastases according to PSMA-PET experience low risk (class I). Patients diagnosed with PSA levels ≥ 0.8 ng/mL and no bone metastases nor visceral metastases prior to MDT were at intermediate risk (class II). PSMA-PET-positive local recurrence and/or pelvic lymph node lesions caused the PSA rise in those two classes. Patients with bone metastases before MDT independently of the PSA value showed a faster relapse (class III). Patients with a PSA level ≥ 0.8 ng/mL, no bone metastases, and the presence of visceral metastases were at very high risk for decreased bRFS (class IV). Table 5 shows the risk classes with the respective bRFS and a description of the prognostic factors. Distribution, duration, and medication of additive ADT use in the risk classes are shown in Table 6.

**Table 5:** Risk classes for bRFS after PSMA-PET-guided RT

Risk class	RPA group	Mean bRFS (95% CI) in months	Median bRFS (95% CI) in months	Risk class characteristics
Class I (low risk)	Group A	36.3 (32.4–40.1)	Not reached	PSA level at PSMA-PET imaging < 0.8 ng/mL, no bone metastases, present local recurrence, and/or pelvic lymph nodes only
Class II (intermediate risk)	Group B	25.8 (22.5–29.1)	25.0 (18.3–31.7)	PSA level at PSMA-PET imaging ≥ 0.8 ng/mL, no bone metastases, no visceral metastases, present local recurrence, and/or pelvic lymph nodes only
Class III (high risk)	Group C	16.0 (12.4–19.6)	16.0 (6.5–25.5)	PSA level at PSMA-PET imaging < 0.8 ng/mL, present bone metastases
	Group D			PSA level at PSMA-PET imaging ≥ 0.8 ng/mL, present bone metastases
Class IV (very high risk)	Group E	5.7 (2.7–8.7)	7.0 (0.6–13.4)	PSA level at PSMA-PET imaging ≥ 0.8 ng/mL, no bone metastases, present visceral metastases

*bRFS*, biochemical relapse-free survival; *PSMA-PET*, prostate-specific membrane antigen-positron emission tomography; *PSA*, prostate-specific antigen; *RPA*, recursive partitioning analysis

**Table 6:** Additive androgen deprivation therapy (ADT) in risk classes

Risk class	Use of ADT (*n*, %)	Median duration of ADT	ADT medication (*n*, %)
Class I (low risk)	31 (27.9)	8 months (range 2–30 months)	LHRH agonist, 18 (58.1) Antiandrogen, 10 (32.3) Unknown, 3 (9.6)
Class II (intermediate risk)	68 (51.1)	6 months (range 1–34 months)	LHRH agonist, 40 (58.8) Antiandrogen, 28 (41.2)
Class III (high risk)	14 (31.1)	8.5 months (range 2–21 months)	LHRH agonist, 8 (57.1) Antiandrogen, 6 (42.9)
Class IV (very high risk)	1 (33.3)	11 months (range 11–11 months)	LHRH agonist, 1 (100.0) Antiandrogen, 0 (0.0)

*ADT*, androgen deprivation therapy; *LHRH*, luteinizing hormone-releasing hormone

## Discussion

The present analysis aimed to develop a prognostic risk classification for bRFS after PSMA-PET-guided MDT for patients with oligorecurrent PC. Three variables emerged as prognostic factors for decreased bRFS in multiple Cox regression: PSA level ≥ 0.8 ng/mL, presence of bone metastases and visceral lesions. With these results, we built a risk classification which showed a convincing internal tenfold cross-validation with 70.6% accuracy.

Recently, few retrospective series with small patient cohorts have demonstrated the feasibility and efficacy of PSMA-PET-guided RT and SBRT [6, 13–18]. Siva et al. prospectively analyzed 33 patients and showed that MDT is a feasible treatment option [19]. In the recent STOMP phase II trial, Ost et al. demonstrated prolonged freedom from ADT with MDT [4]. However, both prospective trials did not use modern PSMA-PET imaging for staging. To our knowledge, the present study is the largest retrospective analysis with patients suffering from oligorecurrent PC treated with PSMA-PET-guided MDT.

Our classification shows that patients with low PSA levels (< 0.8 ng/mL) and pelvic lymph node lesions and/or local recurrence only benefit the most from PSMA-PET-guided MDT (class I). Patients with PSA level ≥ 0.8 ng/mL and pelvic lymph node lesions and/or local recurrence only are at intermediate risk for treatment failure (class II). Hence, our data show a favorable outcome for patients treated with PSMA-PET-guided MDT who are diagnosed with local relapse only. Recently, Schmidt-Hegemann et al. reported a good response to PSMA-PET-guided salvage RT. The authors showed a bRFS of 78% after a median follow-up of 23 months for patients with local relapse only [20]. Furthermore, early treatment of oligorecurrent PC at low PSA levels is desirable. It remains not surprising that the PSA level before MDT is a prognostic factor for biochemical relapse. For local salvage RT, the dictum shifted to “the earlier, the better.” Several studies have shown that a high pre-treatment PSA level before local salvage RT is associated with a decreased biochemical and oncological outcome [21–24]. Early salvage treatment should be initiated at PSA levels of less than 0.5 ng/mL [11]. However, data on the prognostic value of pre-PSA level before MDT in patients with oligorecurrent PC remains rare. Our data showed a pre-treatment PSA level of 0.8 ng/mL as the cutoff value. Although this value seems high, treatment of oligorecurrent PC appears to be beneficial when administered in patients with PSA levels < 0.8 ng/mL or preferably earlier.

Patients with present bone metastases were at higher risk for biochemical relapse (class III). However, those patients still show satisfactory bRFS. Bone metastases are common in patients with metastatic PC [25]. Muacevic et al. reported a local control rate of 95.5% at 24 months in patients treated with robotic SBRT for bone metastases [26]. In a previous study, Habl et al. showed a local progression-free survival (PFS) of 100% at 24 months for patients with bone lesions treated with SBRT. However, the median PSA-PFS was 6.9 months, and the median distant PFS was only 7.4 months [6]. In our study, in-field local control of bone lesions was 100%; bRFS of patients with bone lesions was 16.0 months. The limited effectiveness in systemic control in the series of Habl et al. might be owed to the inability to detect all present metastatic lesions [6] since the authors staged the patients with PET imaging using either the [^68^Ga]PSMA or the [^11^C]choline tracer. Therefore, the less sensitive choline-PET imaging might not have detected all lesions. Although PSMA-PET imaging shows a massive improvement in sensitivity at low PSA levels as compared with choline-PET, the detection rate of recurrences is still approximately 50% at PSA levels below 0.2 ng/mL [27]. Perera et al. reported rates of 58% and 76% for PSA levels of 0.2–1.0 and 1.0–2.0 ng/mL for PET scans with [^68^Ga]PSMA tracers [28]. The optimal threshold value of PSA indicating a high chance of detection of tumor relapse in PSMA-PET imaging remains a topic of discussion.

Patients with visceral metastases experience heavily reduced bRFS (class IV). Although visceral metastases are not common in patients with prostate cancer [25], survival is decreased in general. Gandaglia et al. reported a reduced median overall survival (OS) and PC-specific survival (PSS) in patients with visceral metastases (median OS 16 months, median PSS 26 months) [25]. Only patients with synchronous bone and visceral metastases showed a worse result (median OS 14 months, median PSS 19 months) [29]. The reduced outcome was a reason for defining visceral metastases as high-volume or high-risk disease in the CHAARTED [30] and LATITUDE [31] trial. The data on MDT for patients with visceral metastases is still limited. Ost et al. showed no difference in outcome after MDT between patients with nodal versus non-nodal metastases as visceral lesions [4]. However, the study recruited only one patient with visceral lesions. Although we evaluated a good in-field local control of 100% in patients with visceral metastases, the systemic effect seemed suboptimal. Patients developed biochemical relapse rapidly after local treatment. Therefore, patients with visceral lesions might exhibit a different, more aggressive tumor biology than patients with bone or lymph node metastases. Such patients might benefit from additional ADT to MDT [32]. However, in such cases, MDT should be discussed individually. Bearing in mind the reduced bRFS, palliative ADT alone might be a valid alternative.

Our study has certain limitations. Because of the retrospective nature, the management in terms of treatment was not prospectively defined and differed between institutions. Administration of additive ADT was not standardized and might be a confounder. To account for this problem, we calculated a multiple Cox regression unadjusted and adjusted for additive ADT to show robustness of the model. Furthermore, RT technique and treatment differed between institutions with conventional RT on the one hand to extreme hypofractionated schedules for distant metastasis on the other hand. This must be considered when interpreting the results. However, all visible lesions and thus all the tumor burden was treated with good in-field local control (see Table 2). Therefore, influence on bRFS should be low. The group of patients with visceral metastases was small. Therefore, the results must be interpreted with caution. However, the findings are plausible as several studies show the poor outcome of patients with visceral lesions. The results will be validated externally for further evidence.

## Conclusion

We developed a prognostic risk classification for biochemical relapse after PSMA-PET-guided MDT after RP with convincing internal validation. This classification might be used to weigh the risk-benefit ratio of local curative RT for oligorecurrent lesions. According to this risk classification, patients with PSA levels < 0.8 ng/mL and with local relapse only (local recurrence and/or pelvic lymph nodes) had the most promising bRFS after MDT. PSA levels ≥ 0.8 ng/mL and local relapse only (local recurrence and/or pelvic lymph nodes) indicated intermediate risk for failure. Patients with bone lesions were at higher risk for failure after MDT regardless of the PSA level. However, those patients still show satisfactory bRFS. In patients with visceral metastases, bRFS is heavily decreased; thus, MDT in such cases should be discussed individually.
